# Image inpainting algorithm based on double curvature-driven diffusion model with P-Laplace operator

**DOI:** 10.1371/journal.pone.0305470

**Published:** 2024-07-16

**Authors:** Lifang Xiao, Jianhao Wu

**Affiliations:** 1 School of Computer Science and Engineering, Wuhan Institute of Technology, Wuhan, China; 2 Intelligent Robot Key Laboratory of Hubei Province, Wuhan, China; Islamia University of Bahawalpur: The Islamia University of Bahawalpur Pakistan, PAKISTAN

## Abstract

The method of partial differential equations for image inpainting achieves better repair results and is economically feasible with fast repair time. Addresses the inability of Curvature-Driven Diffusion (CDD) models to repair complex textures or edges when the input image is affected by severe noise or distortion, resulting in discontinuous repair features, blurred detail textures, and an inability to deal with the consistency of global image content, In this paper, we have the CDD model of P-Laplace operator term to image inpainting. In this method, the P-Laplace operator is firstly introduced into the diffusion term of CDD model to regulate the diffusion speed; then the improved CDD model is discretized, and the known information around the broken region is divided into two weighted average iterations to get the inpainting image; finally, the final inpainting image is obtained by weighted averaging the two image inpainting images according to the distancing. Experiments show that the model restoration results in this paper are more rational in terms of texture structure and outperform other models in terms of visualization and objective data. Comparing the inpainting images with 150, 1000 and 100 iterations respectively, Total Variation(TV) model and the CDD model inpainting algorithm always has inpainting traces in details, and TV model can’t meet the visual connectivity, but the algorithm in this paper can remove the inpainting traces well, TV model and the CDD model inpainting algorithm always have inpainting traces in details, and TV model can’t meet the visual connectivity, but the algorithm in this paper can remove the inpainting traces well. Of the images used for testing, the highest PSNR reached 38.7982, SSIM reached 0.9407, and FSIM reached 0.9781, the algorithm not only inpainting the effect and, but also has fewer iterations.

## Introduction

Digital image inpainting is an important part of digital image processing. The final presentation result of digital image inpainting technology must meet the connectivity principle of the human and the fluency of the overall image. On the basis of guaranteeing inpainting of cracks or removal of contaminants, the person or landscape in the inpainting image looks as natural as before.

Image inpainting is the process of restoring missing pixels in digital images in a plausible way. The challenge is that the recovery processes themselves introduce noticeable artifacts within and around the restored image regions. The curvature-driven diffusion model repairs the lost region by diffusing the data near the lost region during image transmission, and uses the edge information of the region to be repaired to diffuse the information outside the region to be repaired into the region to be repaired along the vertical direction of the gradient. The curvature-driven diffusion model introduces a curvature term to make its partial differential equation of higher order, and applies the fast marching method to the boundary of the region to be repaired to gradually advance it from outside to inside, and while advancing it uses the correlation between the pixel to be repaired and its neighboring pixels around it to repair the various discrete pixel points on the boundary. On this basis, the P-Laplace operator is introduced to fill the damaged region by using the performance of the nonlinear anisotropic diffusion of the P-Laplace operator, combining the P-Laplace operator with the diffusion term of the curvature-driven diffusion model to discretize the improved curvature-driven diffusion model model, and dividing the known information around the broken region into two weighted average iterations to obtain the final repair image.

Current restoration methods:

**Deep learning-based image restoration methods**

With the gradual improvement and development of deep learning algorithms, researchers have started to apply deep learning algorithms to the field of image restoration. Researchers have successively proposed Convolutional Neural Networks such as AlexNet, VGG-NET, and ResNet [1–4], which are used to train data to restore images and predict the structure of images efficiently [5–7]. Goodfellow proposed generative adversarial networks [8] in 2014, which consist of a generator and a discriminator, where the generator synthesizes data from a given noise data, and the discriminator discriminates the similarity between the synthesized data and the real data, and if the computationally generated sample image is similar enough to the image of the area to be restored, image restoration can be achieved. With the work of deep learning in the field of image restoration, recurrent neural networks have been applied in the automatic generation of unmarked images, and further research results have been achieved by combining them with convolutional neural networks for image restoration. However, there are the following shortcomings: CNN is widely researched, but there is a shortage for texture restoration; GAN can be applied to image restoration with a large amount of missing data, but the instability problem in the training phase of GAN needs more in-depth research to solve; RNN has excellent performance in processing sequential data, but it is not so good for processing large sample data. Since then, many excellent networks have been proposed. The literature [9] proposed a novel two-stream network for image restoration is proposed, which models structure-constrained texture synthesis and texture-guided structure reconstruction in a coupled manner so that they can better utilize each other for more rational generation. In addition, to enhance global consistency, a bidirectional gated feature fusion (Bi-GFF) module is designed to exchange and combine structural and texture information, and a contextual feature aggregation (CFA) module is developed to refine the generated content affinity learning and multi-scale feature aggregation by region.

In 2023, the literature [10] proposed a U-Net-based latent diffusion model that performs diffusion in a low-resolution latent space while retaining high-resolution information from the original input for the decoding process. Compared to previous latent diffusion models that train VAE-GAN to compress images, the proposed U-Net compression strategy is more stable and can recover highly accurate images without relying on adversarial optimization, aiming to improve the applicability of diffusion models in real-world image recovery. the literature [11] proposed a lightweight single-image super-resolution network that fuses multi-level features. The components are mainly two-level nested residual blocks. To better extract features and reduce the number of parameters, each residual block adopts an asymmetric structure. Moreover, the selection of the appropriate loss function of the neural network requires high hardware requirements, and the network training is time-consuming and other problems. Partial differential equation based image restoration algorithm can avoid these shortcomings.

**Partial differential equation based image restoration algorithm**

The image restoration method of repairing lost areas through known areas originated from the restoration techniques used by craftsmen for damaged artworks, i.e., diffusion restoration based on local information, which mainly revolved around anisotropic filtering until 2000. Malik [12] proposed various anisotropic diffusion equations. These methods can achieve good results in small scratches, small object removal, etc. However, for applications such as restoration of larger areas of broken images, removal of large objects from images, etc., it is difficult to achieve the desired restoration results in these applications.

And essentially, the first consideration in large region restoration is definitely the high-level semantic information of the restored object, and then the repair is based on the large amount of a prior information it has accumulated. For large region restoration, there are mainly two types of effective methods: one is based on texture synthesis techniques [13–17], which can achieve good results in texture detail restoration, but it is difficult to capture the global structure of the image, and the semantics of the image; the other is based on external database search methods [18], which assume that regions surrounded by similar contexts may have similar content, which is very effective when sample images with sufficient visual similarity to the image to be restored can be found, but when the restored image is not well represented in the sample database, false restoration occurs, making the final restoration unsatisfactory.

In 2000, researchers thus proposed early image restoration algorithms based on partial differential equations, which use the idea of diffusion, where the data near the lost region during image transmission arrives to repair the lost region by diffusion, and in 2000 Bertalmio, Sapiro, Caselles, and BallesterIl [19] first proposed a BSCB repair model, which uses the edge information of the region to be repaired to diffuse the information outside the region to be repaired into the region to be repaired along the gradient vertical direction. This process is an iso-illumination line extending along the tangent direction. The algorithm does not require the topology inside the repaired region and is well adapted, but defects such as too slow repair speed and easy blurring after repair hinder its application prospects. In 2001, Chan et al. improved a total variation model(TV) by applying it to image restoration and proposed a new repair model, namely Curvature-Driven Diffusion (CDD) [14], which can overcome the “deficiencies” of TV. It is an extension of the total variance model and the main improvement is the introduction of curvature information in the process of image information diffusion. In 2007, the literature [20] proposed a fast image restoration algorithm for the traditional classical restoration method, which has the disadvantages of slow restoration speed and does not maintain strong edges, by applying a fast marching method to the boundary of the region to be restored. The fast marching method gradually advances from the outside to the inside, and the advancement uses the correlation between the pixel to be repaired and its surrounding neighboring pixels to repair each discrete pixel point on the boundary until the end of the restoration. In the literature [21], the P-Laplace operator is applied to image repair to establish the P-Laplace model for image repair. The model uses the performance of nonlinear anisotropic diffusion of the P-Laplace operator to fill the damaged area, and controls the diffusion direction and time by taking a P value between 1 and 2 to achieve improved repair results and shorter repair time. In 2013, the paper [22] proposed a new tensor diffusion-based wavelet repair model(TDWI) to recover lost or damaged wavelet coefficients. A hybrid model was developed by combining structural adaptive anisotropy regularization with wavelet representation. The shape of the diffusion kernel adaptively changes according to the features of the image, including sharp edges, corner points, and homogeneous regions. Compared with existing wavelet restoration models, this model can more adaptively and accurately control the geometric regularity in the image and has better robustness to noise. In 2016, the literature [23] established a new class of image restoration models based on fractional-order nonlinear anisotropic diffusion equations that employ the P-Laplace norm of the fractional-order gradient of the image intensity function. This model can effectively enhance the texture details of the image and eliminate the step and speckle effect, in addition to effectively removing noise and non-linearly maintaining the high-frequency edges of the image. In 2017, the literature [24] proposed a new TV-Stokes model for image deblurring with a better geometric interpretation. In the image reconstruction, we first calculate the smoothed part of the image from the smoothed tangential field, and then use the anisotropic TV model to obtain the “textured” part of the deblurred image. Solvable properties of the two-step minimization problem are established, and a fast algorithm is given. The new deblurring model is able to capture image details hidden in blurred and noisy images, and the fast algorithm is efficient and robust. In 2018, the literature [25] proposed a wavelet repair model using fractional-order full-variance regularization method. The computational efficiency is improved and the convergence of the new algorithm is ensured. In 2023, the literature [26] proposed a fine inpainting method of incomplete image based on features fusion and two-steps inpainting (FFTI), Firstly, the dynamic memory networks (DMN+) are used to fuse the external features and internal features of the incomplete image to generate the incomplete image optimization map. Secondly, a generation countermeasure generative network with gradient penalty constraints is constructed to guide the generator to rough repair the optimized incomplete image and obtain the rough repair map of the target to be repaired. Finally, the coarse repair graph is further optimized by the idea of coherence of relevant features to obtain the final fine repair graph.

Image repair methods based on partial differential equations can repair small-scale broken images well and can also repair multiple broken fields simultaneously, but the repair results are not satisfactory for images with a large range of missing information, and with the application of image repair in new fields such as biomedicine and face image repair, the quality requirements of the repaired images are getting higher and higher, and the researchers proposed a dynamic weighted matching image restoration algorithm, which better utilizes the known information of the image, improves the image restoration quality, and uses the local average gray-scale entropy fast image restoration algorithm to speed up the computer to perform the restoration of structural information, connect the edges, and later repair the texture components, which can improve the consistency of the image structure, texture, etc.

Embodiment of the advantages of the algorithm in this paper: The algorithm in this paper mainly repairs old photos with scratches and images covered by text. Theoretical analysis and experimental results show that the model has shorter repair time than the CDD model and can obtain visually more natural repaired images.

## CDD inpainting model

**Fig 1:**
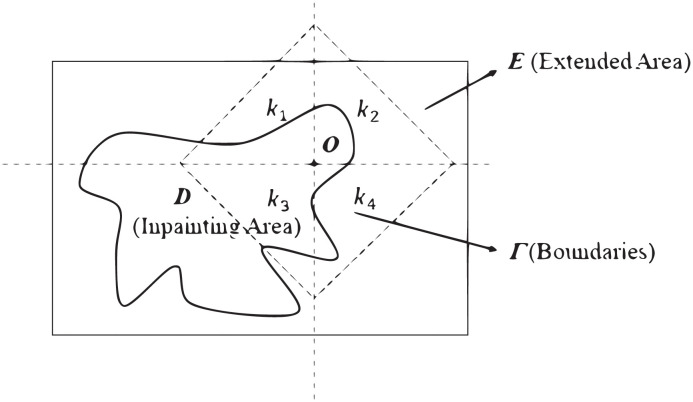
Inpainting domain.

CDD inpainting model is built on TV model. As practiced in the variational methodology, it is very convenient to solve the TV inpainting problem. Fig 1 indicates that *E* (Extended Area), *D* (inpainting Area), etc.

$$\begin{array}{c} J_{\lambda }\left[u\right]=\int_{E\cup D}\left|\nabla u\right|dxdy+\frac{\lambda }{2}\int_{E}{\left|u-u^{0}\right|}^{2}dxdy \end{array}$$ (1)

Where plays the role of the Lagrange multiplier for the constrained variational problem. The Euler-Lagrange equation for the energy functional is

$$\begin{array}{c} \text{div}\;[\frac{\nabla u}{\left|\nabla u\right|}]+\lambda_{e}(u-u^{0})=0 \end{array}$$ (2)

for all, adding Neumann boundary conditions. Here the extended Lagrange multiplier is given by

$$\begin{array}{c} \lambda_{e}\left(o\right)=\{\begin{array}{cc} \lambda , & o\in E\\ 0, & o\in D \end{array} \end{array}$$

The infinitesimal steepest equation for is therefore given by

$$\begin{array}{c} \frac{\partial u}{\partial t}=\nabla (\frac{\nabla u}{\left|\nabla u\right|})+\lambda_{e}(u^{0}-u) \end{array}$$ (3)

Since takes two different values. (2) or (3) is a two-phase problem, and the interface is the boundary P of the inpainting domain. From the numerical point of view, in all of the above differential equations, we replace the curvature term.

Let *D* be an inpainting domain with piecewise smooth boundary, and *E* any fixed closed domain in the complement *D*^*c*^, so that lies in the interior of *E* ∪ *D*. To satisfy the connectivity principle of human, Chan and Shen [15] proposed a new PDE model based on curvature driven diffusions(CDD). Which is closely inspired by the TV inpainting model. The CDD inpainting model is governed by the following PDE:

$$\begin{array}{c} -\nabla [\frac{g(|k|)}{\left|\nabla u\right|}\nabla u]+\lambda_{o}(u-u^{0})=0,\lambda_{o}=\{\begin{array}{c} \lambda ,(x,y)\in E\\ 0,(x,y)\in D \end{array} \end{array}$$ (4)

where k is the scalar curvature $\nabla [\frac{\nabla u}{|\nabla u|}]$. The new ingredient of the CDD model, compared with the TV inpainting model. The choice of a coefficient value of 1 outside the inpainting domain indicates that the model carries out the regular TV denoising task outside *D*. Meanwhile, g(s) can be any appropriate function that penalizes large curvatures and stabilizes small curvatures inside the inpainting domain. In Chan and Shen [15], it is argued that g(s) must satisfy.

$$\begin{array}{c} g(0)=0,g(+\infty )=+\infty \end{array}$$

Thus, for example, one can choose for some *g*(*s*) = *s*^*p*^ for some *p* ≥ 1. Under this condition, the model stretchesout bent level lines inside the inpainting domain, outputs connected objects, and therefore realizes the connectivity principle. For better repair effect, we replace the diffusion function *g*(*s*) with Δ_*p*_*u* where Δ_*p*_*u* = div (|∇*u*|^*p*−2^∇*u*), 1 ≤ *p* < ∞. Using P-Laplace operator to fill the damaged domain can make the image edge transition more natural and improve the inpainting effect. Then, the CDD model with P-Laplace operator is

$$\begin{array}{c} \frac{\partial u}{\partial t}=\text{div}\;[\frac{\left|\Delta_{p}u\right|}{\nabla u}\nabla u]+\lambda_{e}\left(o\right)(u^{0}-u) \end{array}$$ (5)

Using the variational method to solve the extremum question, the following equations are solved

$$\begin{array}{c} \text{div}\;[\frac{\left|\Delta_{p}u\right|}{\nabla u}\nabla u]+\lambda_{e}\left(o\right)(u^{0}-u)=0 \end{array}$$ (6)

## The inpainting algorithm of this paper

Various image inpainting algorithms based on CDD model only make use of the reference information of four neighborhood pixels. Therefore, they cannot keep shape edges and their inpainting precisions high enough. To conquer these difficulties, the image inpainting algorithm based on improved CDD was presented in which the reference information for damaged pixel was extended from 4 into 16 neighborhood pixels. The improved CDD algorithm can effectively improve the inpainting precision and keep shape edeges. Extending the method of repairing damaged points with information from known pixel points in the damaged point domain was inspired by the literature [27], where the CDD model was used to repair damaged points using information from 8 points on *P* = {*N*, *S*, *W*, *E*, *NW*, *NE*, *SW*, *SE*} only. In this paper, we first introduce the P-Laplace operator to modify the diffusion coefficient of the CDD, and then use the improved CDD model to repair the damaged points using the known information of a total of 16 points on p and *p*′ = {*N*, *S*, *W*, *E*, *N*_1_, *S*_1_, *W*_1_, *E*_1_}.

**Fig 2:**
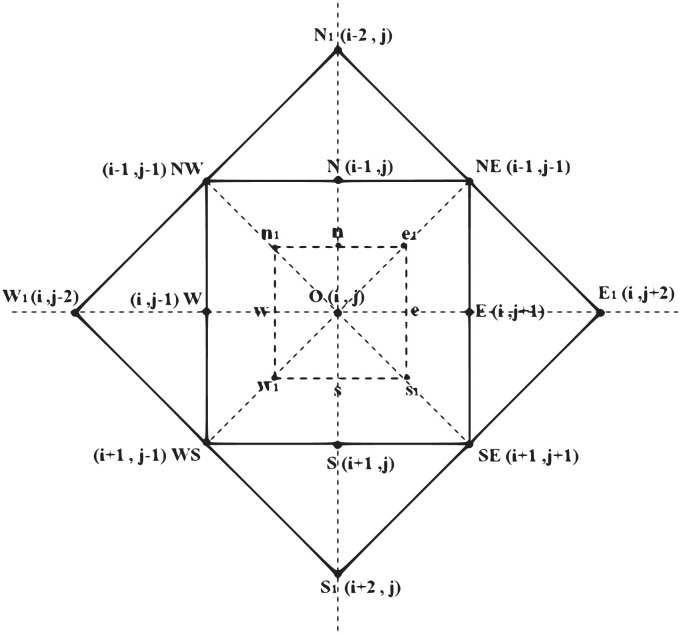
A pixel O and its neighbors.

An improved CDD inpainting algorithm is proposed to inpaint the image twice. Experiments show that the algorithm proposed in this paper not only has good quality of image inpainting, but also the inpainting time takes less, so it is a better algorithm for fast image inpainting. Fig 2 let E, N, W, S denote four adjacent pixels of the pixel O, and e, n, w, s the corresponding four midway points. Write

$$\begin{array}{c} \Lambda_{0}=\left\{E,N,W,S\right\}\;\Lambda_{0}^{\prime }=\left\{e,n,w,s\right\}\;P^{\prime }=N,S,W,E,N_{1},S_{1},W_{1},E_{1} \end{array}$$

Usually, the noise is ignored in the missing or damaged areas. then Eq (3) can be modified as

$$\begin{array}{c} \frac{\partial u}{\partial t}=\text{div}\;[\frac{\left|\Delta_{p}u\right|}{\nabla u}\nabla u] \end{array}$$ (7)

Eq (7) can be simplified as $\frac{\partial u}{\partial t}=\nabla \cdot v$, where $v=(v^{1},v^{2})=\frac{\text{div}({\left|\nabla u\right|}_{z}^{p-2}\nabla u)\nabla u}{{\left|\nabla u\right|}_{\varepsilon }}$, the curvature $k=\text{div}\;({\left|\nabla u\right|}_{\varepsilon }^{p-2}\nabla u)$. We can also use the half-point center difference method to discretize formula (7), as shown in Fig 2.

Then the divergence is first discretized by central differencing:

$$\begin{array}{c} \nabla \cdot v=\frac{\partial v^{1}}{\partial x}+\frac{\partial v^{2}}{\partial y}=\frac{v_{e}^{1}-v_{w}^{1}}{h}+\frac{v_{n}^{2}-v_{s}^{2}}{h} \end{array}$$ (8)

where *h* denotes the grid size, which is always taken to be 1 in image processing. Next, we generate further approximations at the midway points, where image information is not directly available. Take the midpoint e, for example:

$$\begin{array}{c} v_{e}^{1}=\frac{\left|k_{e}\right|}{{\left|\nabla u_{e}\right|}_{\varepsilon }}{\left[\frac{\partial u}{\partial x}\right]}_{e}=\frac{\left|k_{e}\right|}{{\left|\nabla u_{e}\right|}_{\varepsilon }}\frac{u_{E}-u_{O}}{h} \end{array}$$ (9)

$$\begin{array}{c} \nabla u_{e}=(\nabla u_{e}^{1},\nabla u_{e}^{2})=(\frac{u_{E}-u_{O}}{h},\frac{u_{NE}+u_{N}-u_{se}-u_{s}}{4h}) \end{array}$$ (10)

$$\begin{array}{c} {\left|\nabla u_{e}\right|}_{\varepsilon }=\frac{1}{h}\sqrt{{\left(u_{E}-u_{O}\right)}^{2}+{\left[(u_{NE}+u_{N}-u_{SE}-u_{S})/4\right]}^{2}+\varepsilon^{2}} \end{array}$$ (11)

$$\begin{array}{c} k_{e}=\nabla [\frac{\nabla u_{e}}{{\left|\nabla u_{e}\right|}_{\varepsilon }^{2-p}}]=\frac{\partial }{\partial x}[\frac{\nabla u_{e}^{1}}{{\left|\nabla u_{e}^{1}\right|}_{\varepsilon }^{2-p}}]+\frac{\partial }{\partial y}[\frac{\nabla u_{e}^{2}}{{\left|\nabla u_{e}^{2}\right|}_{\varepsilon }^{2-p}}] \end{array}$$ (12)

$$\begin{array}{c} \frac{\partial }{\partial x}[\frac{\nabla u_{e}^{1}}{{\left|\nabla u_{e}\right|}_{\varepsilon }^{2-p}}]=(\frac{\nabla u_{E}^{1}}{{\left|\nabla u_{E}\right|}_{\varepsilon }^{2-p}}-\frac{\nabla u_{O}^{1}}{{\left|\nabla u_{O}\right|}_{\varepsilon }^{2-p}})/h \end{array}$$ (13)

$$\begin{array}{c} \frac{\partial }{\partial y}[\frac{\nabla u_{e}^{2}}{{\left|\nabla u_{e}\right|}_{\varepsilon }^{2-p}}]=(\frac{\nabla u_{NE}^{2}}{{\left|\nabla u_{NE}\right|}_{\varepsilon }^{2-p}}+\frac{\nabla u_{N}^{2}}{{\left|\nabla u_{N}\right|}_{\varepsilon }^{2-p}}\\ -\frac{\nabla u_{SE}^{2}}{{\left|\nabla u_{SE}\right|}_{\varepsilon }^{2-p}}-\frac{\nabla u_{S}^{2}}{{\left|\nabla u_{S}\right|}_{\varepsilon }^{2-p}})/4h \end{array}$$ (14)

$$\begin{array}{c} v_{e}^{1}=\frac{1}{{\left|\nabla u_{e}\right|}_{\varepsilon }}\frac{u_{E}-u_{o}}{h^{2}}[(\frac{\nabla u_{E}^{1}}{{\left|\nabla u_{E}\right|}_{\varepsilon }^{2-p}}-\frac{\nabla u_{o}^{1}}{{\left|\nabla u_{o}\right|}_{\varepsilon }^{2-p}})\\ +\frac{1}{4}(\frac{\nabla u_{NE}^{2}}{{\left|\nabla u_{NE}\right|}_{\varepsilon }^{2-p}}+\frac{\nabla u_{NE}^{2}}{{\left|\nabla u_{NE}\right|}_{\varepsilon }^{2-p}}-\frac{\nabla u_{SE}^{2}}{{\left|\nabla u_{SE}\right|}_{\varepsilon }^{2-p}}-\frac{\nabla u_{S}^{2}}{{\left|\nabla u_{S}\right|}_{\varepsilon }^{2-p}})] \end{array}$$

$$\begin{array}{c} v_{w}^{1}=\frac{1}{{\left|\nabla u_{w}\right|}_{\varepsilon }}\frac{u_{O}-u_{w}}{h^{2}}[(\frac{\nabla u_{W}^{1}}{{\left|\nabla u_{W}\right|}_{\varepsilon }^{2-p}}-\frac{\nabla u_{O}^{1}}{{\left|\nabla u_{O}\right|}_{\varepsilon }^{2-p}})\\ +\frac{1}{4}(\frac{\nabla u_{N}^{2}}{{\left|\nabla u_{N}\right|}_{\varepsilon }^{2-p}}+\frac{\nabla u_{NW}^{2}}{{\left|\nabla u_{NW}\right|}_{\varepsilon }^{2-p}}-\frac{\nabla u_{S}^{2}}{{\left|\nabla u_{S}\right|}_{\varepsilon }^{2-p}}-\frac{\nabla u_{SW}^{2}}{{\left|\nabla u_{SW}\right|}_{\varepsilon }^{2-p}})] \end{array}$$

$$\begin{array}{c} v_{n}^{2}=\frac{1}{{\left|\nabla u_{n}\right|}_{\varepsilon }}\frac{u_{N}-u_{o}}{h^{2}}[(\frac{\nabla u_{N}^{2}}{{\left|\nabla u_{N}\right|}_{\varepsilon }^{2-p}}-\frac{\nabla u_{O}^{2}}{{\left|\nabla u_{O}\right|}_{\varepsilon }^{2-p}})\\ +\frac{1}{4}(\frac{\nabla u_{NE}^{1}}{{\left|\nabla u_{NE}\right|}_{\varepsilon }^{2-p}}+\frac{\nabla u_{E}^{1}}{{\left|\nabla u_{E}\right|}_{\varepsilon }^{2-p}}-\frac{\nabla u_{NW}^{1}}{{\left|\nabla u_{\text{NW}}\right|}_{\varepsilon }^{2-p}}-\frac{\nabla u_{W}^{1}}{{\left|\nabla u_{W}\right|}_{\varepsilon }^{2-p}})] \end{array}$$

$$\begin{array}{c} v_{s}^{2}=\frac{1}{{\left|\nabla u_{s}\right|}_{\varepsilon }}\frac{u_{o}-u_{s}}{h^{2}}[(\frac{\nabla u_{O}^{2}}{{\left|\nabla u_{O}\right|}_{\varepsilon }^{2-p}}-\frac{\nabla u_{S}^{2}}{{\left|\nabla u_{S}\right|}_{\varepsilon }^{2-p}})\\ +\frac{1}{4}(\frac{\nabla u_{E}^{1}}{{\left|\nabla u_{E}\right|}_{\varepsilon }^{2-p}}+\frac{\nabla u_{SE}^{1}}{{\left|\nabla u_{SE}\right|}_{\varepsilon }^{2-p}}-\frac{\nabla u_{W}^{1}}{{\left|\nabla u_{W}\right|}_{\varepsilon }^{2-p}}-\frac{\nabla u_{SW}^{1}}{{\left|\nabla u_{SW}\right|}_{\varepsilon }^{2-p}})] \end{array}$$

Similar discussion applies to the other three directions N, W, S and the other half-pixel points n, w, s. Therefore, at a pixel O, the Eq (2) is discretized to:

$$\begin{array}{c} \sum_{P\in \Lambda_{0},d\in \Lambda_{0}^{\prime }}\frac{\left|k_{d}\right|}{\left|\nabla u\right|}(u_{O}-u_{P})+\lambda_{o}(u_{O}-u_{O}^{0})=0 \end{array}$$ (15)

where the function $u_{O}^{0}$ represents the original defective image. For any arbitrary point $d\in \Lambda_{0}^{\prime }$, the function *w*_*d*,*ε*_ be defined:

$$\begin{array}{c} w_{d,\varepsilon }=\frac{\left|k_{d}\right|}{{\left|\nabla u_{d}\right|}_{\varepsilon }}=\frac{\left|k_{d}\right|}{\sqrt{{\left|\nabla u_{d}\right|}^{2}+\varepsilon^{2}}} \end{array}$$ (16)

where the parameter *ε* > 0. When *ε* = 0, the function *w*_*d*_ is simplified as:

$$\begin{array}{c} w_{d}=\frac{\left|k_{d}\right|}{\left|\nabla u_{d}\right|} \end{array}$$ (17)

Similar, the gradient norma |∇*u*_*w*_|, |∇*u*_*n*_|, |∇*u*_*s*_| of other half-pixel points can be calculated. We solve Eq (15) to obtain

$$\begin{array}{c} u_{o}=\frac{\sum_{P\in A_{0},d\in \Lambda_{0}^{\prime }}\frac{\left|k_{d}\right|}{\left|\nabla u_{d}\right|}u_{P}}{\sum_{d\in \Lambda_{0}^{\prime }}\frac{\left|k_{d}\right|}{\left|\nabla u_{d}\right|}+\lambda_{o}}+\frac{\lambda_{o}}{\sum_{d\in \Lambda_{0}^{\prime }}\frac{\left|k_{d}\right|}{\left|\nabla u_{d}\right|}+\lambda_{o}}u_{o}^{0} \end{array}$$ (18)

The formula $\frac{\left|k_{d}\right|}{\left|\nabla u_{d}\right|}$ represents the diffusion coefficient corresponding to the four pixels e, n, w, s. The function *u*_*p*_ represents the gray value of the four points:E, N, W, S. Let

$$\begin{array}{c} w_{d}=\frac{\left|k_{d}\right|}{\left|\nabla u_{d}\right|},h_{Od}=\frac{w_{d}}{\sum_{d\in \Lambda_{0}^{\prime }}w_{d}+\lambda_{o}},h_{OO}=\frac{\lambda_{o}}{\sum_{d\in \Lambda_{0}^{\prime }}w_{d}+\lambda_{o}} \end{array}$$ (19)

Using formula (19) to simplify formula (18), a new inpainting formula is obtained:

$$\begin{array}{c} u_{O}=\sum_{P\in \Lambda_{0},d\in \Lambda_{0}^{\prime }}h_{Od}u_{P}+h_{OO}u_{o}^{0} \end{array}$$ (20)

With

$$\begin{array}{c} \sum_{P\in \Lambda_{0}}h_{op}+h_{oo}=1 \end{array}$$

Eq (20) is in the form of a low pass filter, which is of course a system of nonlinear equations since the filter coefficients all depend on u. Freezing the filter coefficients (to linearize the equations), and adopting the Causs-Jacobi iteration scheme for linear systems, at each step n, we update *u*^(*n*−1)^ to *u*^(*n*)^ by

$$\begin{array}{c} u_{O}^{n}=\sum_{P\in \Lambda_{0},d\in \Lambda_{0}^{\prime }}h_{Od}^{n-1}u_{P}^{n-1}+h_{OO}^{n-1}u_{O}^{n-1} \end{array}$$ (21)

Where *h*^*m*−1^ = *h*(*u*^*m*−1^). Since *h* is a low pass filter, the iterative algorithm is stable and satisfies the maximum principle [16]. In particular, the gray value interval [0, 1] is always preserved during the iterating process.

Useful variations of the algorithm can be obtained by altering the definition *w*_*d*_ in (17). Experiments show that such variations sometimes work better for inpainting sharp edges in the digital setting. In implementation, the weights *w*_*d*_ are

$$\begin{array}{c} w_{d}=\frac{\left|k_{d}\right|}{{\left|\nabla u_{d}\right|}_{\varepsilon }}=\frac{\left|k_{d}\right|}{\sqrt{\varepsilon^{2}+{\left|\nabla u\right|}^{2}}} \end{array}$$ (22)

For some small number *ε*, to avoid a zero divisor in smooth regions. Notice that choosing a large *ε* brings the TV model closer to the harmonic inpainting(especially computationally, since the spatial step size *h* is set to 1, and *u* takes values from the finite gray-scale interval [0.1]). In addition, as *ε* gets bigger, the convergence the iteration scheme speeds up.

Although the pixels in area *E* are known, the iterative operation is still performed. The new value of the point *O* is calculated from the old value and the adjacent pixels. The iterative operation is performed for each pixel in the area *E*, so that the known information is gradually advaced to the damaged area, which inpaints the image.

The above analysis shows that the restoration value of the point *O* is only related to eight pixels in its area on the surface, but in fact, every pixel in the area participates in the iterative operation, and the restoration value of the point *O* is related to the pixel value in the area. However, because each operation only considers eight pixels in the field of point *O*, the information obtained is limited.

In order to reduce the inpainting time, our algorithm uses a weighted average kernel with the diffusion coefficient to inpaint the highest bit layer of image.

At the highest level of the image, we use the new inpainting formula (20) to calculate the inpainting point *O* by eight pixels neighborhood of the point *O*, and use the information of the eight points to predict the point *O*. When |∇*u*_*d*_| = 0, the weighting coefficient tends to infinity, So the adjustment parameter *ε* is introduced. The following inpainting formula is obtained:

$$\begin{array}{c} u_{0}=\frac{\sum_{P\in \Lambda_{0},d\in \Lambda_{0}^{\prime }}w_{d,\varepsilon }u_{P}}{\sum_{d\in \Lambda_{0}^{\prime }}w_{d,\varepsilon }} \end{array}$$ (23)

As can be seen from the above equation, the inpainting formula is closely related to the curvature term and gradient. When the gradient is large, the weighting coefficient is small, and when the gradient is small, the weighting coefficient is large, so the inpainting algorithm is also anisotropic. Due to the addition of gradient and curvature terms, the “connectivity criterion” can be satisfied.

The essence of the CDD model is to use the weighted average of four points in the field of points to be repaired as the change variable in the repair iterative process. This model can achieve both denoising and preserving image edge in the process of restoration. However, it only uses 8 points{N, S, W, E, NE, NW, SW, SE}in the field of points to be repaired. If four more points in the repair area are added to provide effective information for the points to be repaired, the accuracy of the repaired image will be greatly improved. Following this idea, on the basis of the CDD model, the solution information of the points to be repaired is extended to 12 points in the neighborhood, so that the accuracy of the repaired images can be improved under the condition of increasing the time complexity. Then 8 points in the repair area are selected again, and the repair points are repaired by using the CDD model, and a formula (24) was obtained,

$$\begin{array}{c} u_{O}^{\prime }=\frac{\frac{2}{h^{2}}\sum_{P\in \Lambda_{0},d\in \Lambda_{0}^{\prime }}\frac{\left|k_{d}\right|}{\left|\nabla u_{d}\right|}u_{P}}{\sum_{d\in \Lambda_{0}^{\prime }}\frac{\left|k_{d}\right|}{\left|\nabla u_{d}\right|}+\lambda_{o}}+\frac{\lambda_{o}}{\sum_{d\in \Lambda_{0}^{\prime }}\frac{\left|k_{d}\right|}{\left|\nabla u_{d}\right|}+\lambda_{o}}u_{O}^{0} \end{array}$$ (24)

where, Λ_0_ = {*E*_1_, *N*_1_, *W*_1_, *S*_1_}, $\Lambda_{0}^{\prime }=\left\{e_{1},n_{1},w_{1},s_{1}\right\}$.

Formula (24) is obtained by rotating the field points of Formula (18) in the following way:

$$\begin{array}{c} E\to NE,N\to NW,W\to WS,S\to SE,\\ SE\to E_{1,}NE\to N_{1},NW\to W_{1},SW\to S_{1} \end{array}$$

In the above process, the repair points have been repaired twice at the same time and the results obtained are respectively *u*_0_ and $u_{0}^{\prime }$. Below, the weighted average of these two different results is carried out to obtain the final repair effect.

Since two different fields with different distances of the point *O* to be repaired are used respectively in function *u*_0_ and $u_{0}^{\prime }$.

Therefore, the difference step size used to solve *O* is different, which results in different influence on *O*. Therefore, according to the distance ratio and weighted average, the final repair value of the points to be repaired is as follows:

$$\begin{array}{c} u=\frac{1}{1+\sqrt{2}}u_{0}^{\prime }+\frac{\sqrt{2}}{1+\sqrt{2}}u_{0} \end{array}$$ (25)

Specific details and methods:

1. Firstly, based on the information of the eight points {*N*, *E*, *S*, *W*, *NE*, *SE*, *WS*, *NW*} of the inner solid square in Fig 2, the point *O* to be repaired is repaired using Formula (18) to obtain *u*_0_.
2. Then, based on the information of the eight points {*E*_1_, *N*_1_, *W*_1_, *S*_1_, *NE*, *SE*, *WS*, *NW*} of the external solid square in Fig 2, the point *O* to be repaired is repaired using Formula (24) to obtain $u_{0}^{\prime }$.
3. Finally, the repair values *u*_0_ and $u_{0}^{\prime }$ are superimposed by taking the weights according to the degree of influence of the distance, as in Formula (25), to obtain the value of the point to be repaired *O*, *u*.

Reasons for empowerment:

1. The closer it is to the point to be repaired, the greater its correlation with the point to be repaired, and the greater its influence on the point to be repaired;
2. The image connectivity criterion is satisfied. For a certain pixel related to the repair point, when the distance of unit length is increased, the increased pixel value is equal. Guass-Jacobi iterative method is adopted:
  $$\begin{array}{c} u^{\left(n\right)}=\frac{1}{1+\sqrt{2}}u_{0}^{\left(n-1\right)}+\frac{\sqrt{2}}{1+\sqrt{2}}u_{0}^{\left(n-1\right)} \end{array}$$ (26)

### Algorithm implementation steps

**Step**1:Read the damaged image. Read the damaged image through the Imread function that comes from *MATLAB*. After reading, the bit image layering technology is used for layering. Take the highest bit layer of image after layering and enter the next step.

**Step**2: Determine the damaged area *D* of the image. The image after layering needs to be further processed, and the mask marking method is used to separate the damaged area from the known area. Mark the damaged area with a mask, and set the mask value of the pixel point in the damaged area to zero, it means that there is the damaged point and must be inpainted. Let the mask value within the known range [1, 255], it means that this pixel point is known and does not need to inpaint. The determined damaged area must be participated by humans, and human-computer interaction can confirm the entire damaged area.

**Step**3:Find the damaged area and inpaint it. The image inpainting process is the process of predicting unknown points from known points. Therefore, before inpainting, it is necessary to find the boundary of the known area, and start the iterative operation step by step from the boundary to the interior.

**Step**4:Take the image of the damaged area and use the improved CDD model to restore. After restoring, it is time to go to the second step and continue to use the improved CDD model to inpaint the image of the next damaged area.

**Step**5:Perform image reconstruction. Finally the restored image can be obtained, and the image is output.

**Algorithm 1**: The Curvature-Driven Diffusion model with the P-Laplace operator term.

**1** **Step1**:

**2** function newcdd(u);

 **Input**: Image to be inpainting *u*^*o*^

 **Output**: Image of completed inpainting u

**3** $[u_{x}^{o},u_{y}^{o}]=\text{gradient}(u^{o})$ ;

**4** $K_{e}=\frac{\partial U_{e}^{o}}{\partial x}+\frac{\partial U_{e}^{o}}{\partial y}$ , $K_{w}=\frac{\partial U_{w}^{o}}{\partial x}+\frac{\partial U_{w}^{o}}{\partial y}$, $K_{s}=\frac{\partial U_{s}^{o}}{\partial x}+\frac{\partial U_{s}^{o}}{\partial y}$, $K_{n}=\frac{\partial U_{n}^{o}}{\partial n}+\frac{\partial U_{n}^{o}}{\partial y}$,;

**5** $U_{e}={\left|\nabla u_{e}^{o}\right|}_{\varepsilon }$ , $U_{w}={\left|\nabla u_{w}^{o}\right|}_{\varepsilon }$, $U_{s}={\left|\nabla u_{s}^{o}\right|}_{\varepsilon }$, $U_{n}={\left|\nabla u_{n}^{o}\right|}_{\varepsilon }$;

**6** *ω*_*e*_=*K*_*e*_/sqrt(1+*U*_*e*_)+1,*ω*_*w*_=*K*_*w*_/sqrt(1+*U*_*w*_)+1

 *ω*_*s*_=*K*_*s*_/sqrt(1+*U*_*s*_)+1,*ω*_*n*_=*K*_*n*_/sqrt(1+*U*_*n*_)+1

**7** $u=(\omega_{e}u_{e}^{o}+\omega_{w}u_{w}^{o}+\omega_{s}u_{s}^{o}+\omega_{n}u_{n}^{o})/(\omega_{e}+\omega_{w}+\omega_{s}+\omega_{n})$

**8 Step2**:

**9** function newcdd(*u*′);

 **Input**: Image to be inpainting *u*^*o*^

 **Output**: Image of completed inpainting *u*′

**10** $\left[u_{x}^{o},u_{y}^{o}]=\text{gradient}(u^{o}\right)$ ;

**11** $K_{e}=\frac{\partial U_{e_{1}}^{o}}{\partial x}+\frac{\partial U_{e_{1}}^{o}}{\partial y}$ , $K_{w}=\frac{\partial U_{w_{1}}^{o}}{\partial x}+\frac{\partial U_{w_{1}}^{o}}{\partial y}$, $K_{s}=\frac{\partial U_{s_{1}}^{o}}{\partial x}+\frac{\partial U_{s_{1}}^{o}}{\partial y}$, $K_{n}=\frac{\partial U_{n_{1}}^{o}}{\partial n}+\frac{\partial U_{n_{1}}^{o}}{\partial y}$,;

**12** $U_{e_{1}}={\left|\nabla u_{e_{1}}^{o}\right|}_{\varepsilon }$ , $U_{w_{1}}={\left|\nabla u_{w_{1}}^{o}\right|}_{\varepsilon }$, $U_{s_{1}}={\left|\nabla u_{s_{1}}^{o}\right|}_{\varepsilon }$, $U_{n_{1}}={\left|\nabla u_{n_{1}}^{o}\right|}_{\varepsilon }$;

**13** $\omega_{e_{1}}=K_{e}/\text{sqrt}(1+U_{e})+1,\omega_{w_{1}}=K_{w}/\text{sqrt}(1+U_{w})+1$

$\omega_{s_{1}}=K_{s}/\text{sqrt}(1+U_{s})+1,\omega_{n_{1}}=K_{n}/\text{sqrt}(1+U_{n})+1$

$u^{\prime }=(\omega_{e_{1}}u_{e_{1}}^{o}+\omega_{w_{1}}u_{w_{1}}^{o}+\omega_{s_{1}}u_{s_{1}}^{o}+\omega_{n_{1}}u_{n_{1}}^{o})/(\omega_{e}+\omega_{w}+\omega_{s}+\omega_{n})$

**Step3**:

 **Input**: Image to be inpainting *u*, *u*′

 **Output**: Image of completed inpainting I

**16** $I=\frac{1}{1+\sqrt{2}}u^{\prime }+\frac{\sqrt{2}}{1+\sqrt{2}}u$

## Experimental results

We have implemented the algorithm with MatlabR2016a on the PC with i5-1035G1, 1.00GHz, 16G memory. The size of the image used is 256 * 256. Take the parameters λ = 1, *P* = 1.6 and carry out experiments on large spots and scratches respectively.

In order to verify the experimental results, we use TV model, CDD model and the improved algorithm to carry out restoration experiments. The algorithm parameter *ε* = 1. The signal-to-noise ratio is calculated as follows:

$$\begin{array}{c} PSNR=10\text{lg}(\frac{\sum_{i=1}^{M}\sum_{j=1}^{N}{\left(u\left(i,j\right)\right)}^{2}}{\sum_{i=1}^{N}\sum_{j=1}^{N}{\left(u_{0}(i_{,},j)-u\left(i,j\right)\right)}^{2}}) \end{array}$$ (27)

**Fig 3:**
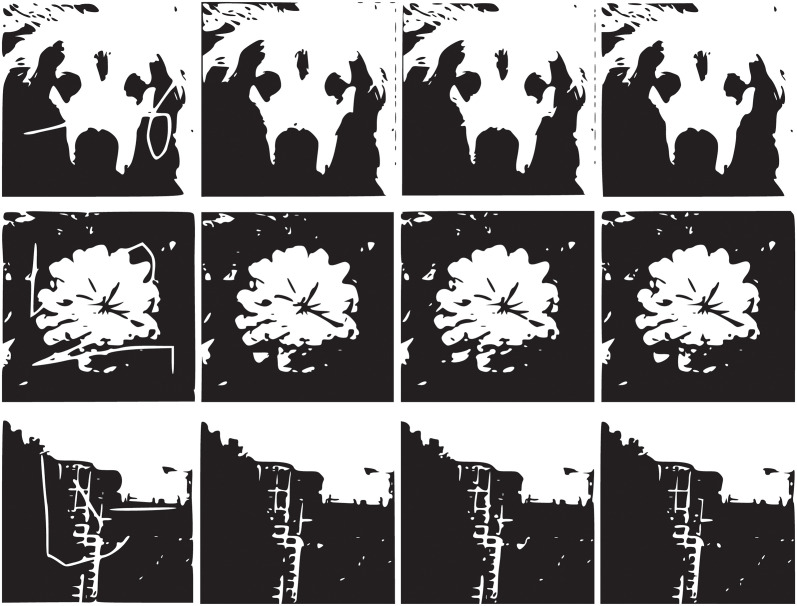
Image inpainting of dog, flower and buildings.

Considering the importance of inpainting application, several metrics have been proposed in the literature [28] specially dedicated to the image inpainting quality assessment. Then, we apply Structure Similarity Index Measure(SSIM), Feature Similarity Index Mersure (FSIM) and Normalized Mean Square Error(NMSE)to evaluate the quality of the images:

$$\begin{array}{c} \text{SSIM}\left(x,y\right)=\frac{\left(2\mu_{x}\mu_{y}+C_{1})(2\sigma_{xy}+C_{2}\right)}{\left(\mu_{x}^{2}+\mu_{y}^{2}+C_{1})(\sigma_{x}^{2}+\sigma_{y}^{2}+C_{2}\right)} \end{array}$$ (28)

$$\begin{array}{c} FSIM=\frac{\sum_{x\in \Omega }S_{L}\left(x\right)PC_{m}\left(x\right)}{\sum_{x\in \Omega }PC_{m}\left(x\right)} \end{array}$$ (29)

$$\begin{array}{c} \text{NMSE}=\frac{\sum_{i=1}^{M}\sum_{j=1}^{N}{\left[g\left(i,j\right)-\hat{g}\left(i,j\right)\right]}^{2}}{\sum_{i=1}^{M}\sum_{j=1}^{N}{\left[g\left(i,j\right)\right]}^{2}} \end{array}$$ (30)

where M = N = 255. The above images are all taken by ourselves. For Fig 3, for the original image, TV model and CDD model inpainting algorithm are basically consistent with the inpainting results of the algorithm in this paper. In Fig 3, the number of iterations is 150,1000,100 respectively, CDD model and TV model can clearly show up the inpainting traces in the inpainting results, but the inpainting traces are almost invisible with the algorithm in this paper. The algorithm in this paper is least iterations, and the restoration effect of the restoration algorithm in this paper is best.

**Fig 4:**
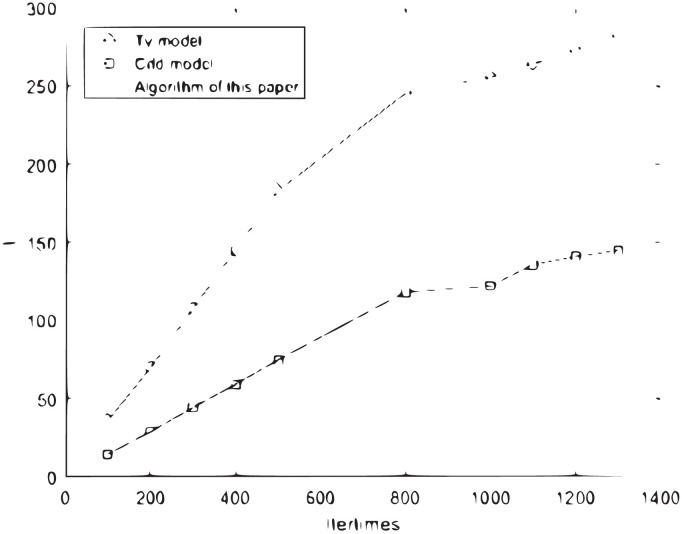
Iteration times and time curve.

The above three images of dogs, flowers and buildings have been studied by TV model, CDD model and the algorithm proposed in this paper. From Tables 1–3 and Fig 4, it can be seen that there are obvious traces of restoration in TV and CDD restored images, and the restoration effect of the restoration algorithm proposed in this paper is better. In the image inpainting experiments, compared with the TV model, the algorithm proposed in this paper consumes the least time and has the best effect, so the algorithm proposed in this paper is better than the TV model and CDD model. Fig 4 shows graphically that the iteration time growth rate of the proposed algorithm and TV model is much larger than that of the CDD model with the growth of time.

**Table 1:** Comparison of algorithm time (s) and signal-to-noise ratio (PSNR).

Image	TV model		CDD model		proposed algorithm	
	Time	PSNR	Time	PSNR	Time	PSNR
Fig 3 *Dog*	282.9851	22.8323	144.9940	30.0024	228.6431	38.7982
Fig 3 *Flower*	273.2293	21.1838	141.1240	29.5230	224.5521	37.8147
Fig 3 *Buildings*	264.4490	20.3567	135.8070	28.4462	219.9650	36.3820

**Table 2:** Comparison of Structure Similarity Index Mersure(SSIM) and Feature Similarity Index Mersure (FSIM).

Image	TV model		CDD model		proposed algorithm	
	SSIM	FSIM	SSIM	FSIM	SSIM	FSIM
Fig 3 *Dog*	0.7618	0.8897	0.8763	0.9013	0.9211	0.9781
Fig 3 *Flower*	0.8183	0.8613	0.8972	0.9345	0.9407	0.9579
Fig 3 *Buildings*	0.8612	0.8496	0.8492	0.8515	0.9323	0.9650

**Table 3:** Comparison of Normalized Mean Square Error(NMSE).

Image	TV model	CDD model	proposed algorithm
	NMSE	NMSE	NMSE
Fig 3 *Dog*	0.01667	0.0204	0.0151
Fig 3 *Flower*	0.0193	0.0307	0.0175
Fig 3 *Buildings*	0.0186	0.0267	0.1661

The image restoration model based on partial differential equations utilizes useful information around the area to be repaired to diffuse into the area. The advantage of this method is its simplicity of use. However, this method cannot repair large damaged areas, while neural network image repair methods can repair large damaged areas. In reference [29], the occlusion of facial images reached 25%, and the PSNR value after repairing using CE network reached 30.75. Although neural networks require high software and hardware conditions, the method of repairing images using neural networks has a better effect on repairing areas with large damage. In the future, neural network methods will be the mainstream method for image processing.

## Conclusions

On the basis of studying TV model and CDD model inpainting algorithm proposed by Chan et al., the improved algorithm which uses an improved CDD model to perform on the highest level of image. The algorithm uses the structural information such as image gradient and curvature to inpaint, and achieves good results. In the case of fewer iterations, the improved algorithm is better than TV model, CDD model, and the image texture features can also be inpainted. It is worth improving that because the algorithm in this paper targets grayscale images, when there is a brighter area in the image, it will default to blank for repair, causing the area to become blurred and destroy the original connectivity. Moreover, when there are a large number of blank areas, the repair effect is not satisfactory. The above problems deserve more in-depth research.

Overall, the design of deep learning network and the selection of loss function during training are to be explored, and the selection of appropriate loss function will improve the image repair quality while also speeding up the training speed of deep learning. The improvement of image restoration quality can also be solved by reducing the depth of the noise model. Therefore, more in-depth research is needed on how to design a more perfect denoising model and how to design a restoration network with universal applicability to improve the accuracy of the restoration results.
